# Types of social capital resources and self-rated health among the Norwegian adult population

**DOI:** 10.1186/1475-9276-9-8

**Published:** 2010-03-17

**Authors:** Abdi A Gele, Ivan Harsløf

**Affiliations:** 1 Research Group for Inclusive Social Welfare Policies, The Department of Social Science, Oslo University College, Oslo, Norway; 2 Section for International Health, Department of General Practice and Community Medicine, University of Oslo, Oslo, Norway

## Abstract

**Background:**

Social inequalities in health are large in Norway. In part, these inequalities may stem from differences in access to supportive social networks - since occupying disadvantaged positions in affluent societies has been associated with disposing poor network resources. Research has demonstrated that social networks are fundamental resources in the prevention of mental and physical illness. However, to determine potentials for public health action one needs to explore the health impact of *different types *of network resources and analyze if the association between socioeconomic position and self-rated health is partially explained by social network factors. That is the aim of this paper.

**Methods:**

Cross-sectional data were collected in 2007, through a postal survey from a gross sample of 8000 Norwegian adults, of which 3,190 (about 40%) responded. The outcome variable was self-rated health. Our main explanatory variables were indicators of socioeconomic positions and social capital indicators that was measured by different indicators that were grouped under '*bonding'*, '*bridging' *and '*linking' *social capital. Demographic data were collected for statistical control. Generalized ordered logistic regression analysis was performed.

**Result:**

Results indicated that those who had someone to talk to when distressed were more likely to rate their health as good compared to those deprived of such person(s) (OR: 2.17, 95% CI: 1.55, 3.02). Similarly, those who were active members in two or more social organisations (OR: 1.73, 95% CI: 1.34, 2.22) and those who count a medical doctor among their friends (OR: 1.51, 95% CI: 1.13, 2.00) report better health. The association between self-rated health and socio-economic background indicators were marginally attenuated when social network indicators were added into the model.

**Conclusion:**

Among different types of network resources, close and strong friendship-based ties are of importance for people's health in Norway. Networks linking people to high-educated persons are also of importance. Measures aiming at strengthening these types of network resources for socially disadvantaged groups might reduce social inequalities in health.

## Background

Social inequality in health has been a major public health concern in many European countries [1]. In Norway, health inequalities - whether measured in relative or absolute terms - are remarkably large and persistent despite strong redistributive welfare policies [2]. The immediate concern is, thus, the identification of the determinants of health disparities and the concurrent development of policies to reduce them [3].

A growing bulk of evidence suggests that public health strategies that strengthen people's social networks, or 'social capital', may have considerable potentials for health improvement, particularly for the most disadvantaged groups in society [4-7]. Social capital is taken to represent interpersonal support systems that may, among other benefits, be helpful in matters of personal health and community health action [8,9]. The basic elements of what has later been conceptionalised as social capital was first examined by Durkheim when he studied social influences on suicide [10]. Within the social sciences two main strands building on the notion of social capital have evolved [11]. One strand approaches the subject mainly from a macro perspective, assessing the social capital of communities in terms of shared identity and interests, trust, extent of collaboration etc. Another strand, adopts a micro perspective, assessing individuals' social capital in terms of the network resources they command. However, the prominent role assigned to personal network connections seems to cut across different perspectives on social capital [12-14].

In Norway, an important determinant of social inequalities in health might originate from differences in access to supportive social network resources as constituted by relations to family, friends and acquaintances. Cross-national comparative research has demonstrated that occupying a disadvantaged position in affluent societies is associated with a low degree of social integration in personal networks and formal associations [15]. Having such low degree of social integration may deprive disadvantaged groups of the social network resources of importance for their psychological and physical health [16,17].

There is a growing body of evidence supporting the idea that social network ties provide important psychological health resources for the prevention of mental and physical illness, as well as for the promotion and restoration of general health [16,17]. Findings suggest that social network ties improve health conditions [18,19] and health-related measures of the quality of life [20], spread happiness [21] and improve the health of people with chronic diseases such as diabetes [22], while it reduces distress [5]. Experimental studies involving both animal and human subjects have shown that socially isolated individuals have heightened cardiovascular reactivity, which has been linked to atherosclerosis [23,24]. Evidence shows that mortality rates increase with lack of social relationships [25]. For example, a study in France found almost a three-fold higher risk of mortality for older adults with few network connections than their less isolated counterparts [26].

The growing evidence connecting social network ties to health has generated a debate regarding *the way in which *social network ties affect health. Few mechanisms have been repeatedly cited in the literature. Among them are psychological mechanisms, including the effect of being integrated into social networks on feelings of self-esteem and the ability to cope [27], and behavioral mechanisms, including the social impact and social regulation of health behaviors by people within the network, and the health benefits of social engagement and participation [4].

There appears to be a consensus in the literature that one can distinguish at least three main types of social support to access through networks: *emotional *(providing intimacy, attachment, caring, and concern), *informational *(providing advice, guidance, or information relevant to the situation), and *instrumental *(providing aid or assistance) [28,29]. Furthermore, it has been suggested that being embedded in different network structures are in different ways instrumental in providing these types of support. First, networks characterized by strong, long-lasting relationships between people of equal social standing are supposedly best able to provide emotional support. Such types of networks are therefore said to represent *bonding social capital*. According to Woolcock, bonding social capital should denote ties between people in similar situation such as immediate family, close friends and neighbors [30]. Secondly, networks characterized by complex and fluctuating contacts between people from different social environments are supposedly best able to provide informational support due to its dynamic and diversified nature. Such types of networks are said to represent *bridging social capital*. *Bridging social capital *encompasses more distant ties of like persons, such as loose friendships and workmates [30]. Finally, networks characterized by contacts between people from different social strata or positions of formal authority are supposedly best able to provide instrumental support. Such types of networks are said to represent *linking social capital*. Szreter and Woolcock emphasize that such contacts in order to come under the notion of this type of social capital should cut across 'explicit, formal, or institutionalized power or authority gradients in society'[31].

There are indisputable evidences on the association between social network resources and self-reported health. However, it is imperative to continue distinguishing between different types of social capital resources, theoretically and empirically, as they might imply different kinds of resources, influence, support, and obligations that are crucial for people's health. To isolate the social determinants of health, more works that explore the importance of network resources accommodated by these different subtypes of social capital are needed. The present study utilized a large sample of the Norwegian adult population, and employed indicators of bonding, bridging and linking social capital pertaining to individuals' networks to assess the impact of these dimensions on the association between socioeconomic position and self-rated health.

## Methods

The data was collected through a postal survey in the spring of 2007. The data collection was a part of the larger study 'Living conditions and social networks', a collaboration between Norwegian Social Research (NOVA), Fafo Institute for Labour and Social Research and Oslo University College (OUC). To accommodate the broad target population, the wording of the questionnaire was reviewed carefully by an external panel of survey experts. Furthermore, the framing of questions on access to network resources and other key issues was tested on two focus groups consisting of students and administrative staff at OUC. Statistics Norway drew a representative random sample from the National Population Register (sampling frame) and fielded the survey. Out of a gross sample of 8,000 Norwegian adults between the age 18 and 74 years, 3,190 people filled the questionnaires, constituting a response rate of about 40%.

Comparing the study sample to the general population, as done in Table 1, shows that the data is representative when it comes to gender and place of residence (at county level). When it comes to ethnicity, people of immigrant backgrounds are slightly underrepresented. Moreover, unemployed persons appear to be highly underrepresented. However, information on immigrant background, employment status and educational level included in the multivariate models adjusted for these drawbacks of the study sample.

**Table 1:** A comparison of the demographic characteristics found in the general Norwegian population and in the study sample

Characteristics	General population		Study sample	
	
	**No**.	%	**No**.	%
**Gender**^a^				
Female	1 829 454	50.5	1 612	50.5
Male	1 866 317	49.5	1 578	49.5
*Total*	3 695 771	100	3 190	100
**County**^b^				
Oslo-Akershus	981 000	22	779	24.4
Hedmark-Oppland	368 000	8.2	267	8.4
Sør-Østlandet	869 000	19.4	571	17.9
Agder- Rogaland	633 000	14	427	13.4
Vestlandet	784 000	17.5	571	17.9
Trøndelag	391 000	8.7	281	8.8
Nord-Norge	459 000	10.2	294	9.2
*Total*	4 485 000	100	3 190	100
**Ethnicity**^a^				
Non-immigrants	4 025 000	89.7	2 950	92.5
Immigrants	460 000	10.3	227	7.1
*Total*	4 485 000	100	3 177	99.7
**Occupational status in 2005**^c^				
Employed/self-employed	1 607 000	83.2	2430	76.2
Unemployed	67 000	4	40	1.3
Outside labour force	^d^	^d^	699	21.9
*Total*	1 674 000	87.2	3169	99.3

^a) ^The figures for the general population are from 2009 (source: Statistics Norway, databank)

^b) ^The figures for the general population are from 2001 (Source: Statistics Norway, Population and housing census 2001 [56]

^c) ^The figures for occupational status are confined to people 25-54 years of age for both the general population and the study sample. The source for the general population is Statistics Norway databank and refers to 2005. The source for the study population is administrative data from 2005 provided by Statistics Norway.

^d) ^No directly comparable info.

The outcome variable was self-assessed health, measured by a single item: 'How will you describe your current health condition?' The answer categories were: 'Very good', 'good', 'fair', 'bad' and 'very bad'. The variable was recoded into three categories ('3' = 'very good'/'good', '2' = 'fair' and '1' = 'bad'/'very bad').

Our main explanatory variables for predicting self-assessed health were indicators of socioeconomic position and indicators of different types of network resources.

To assess socioeconomic position we used level of education and peoples' relation to the labor market. This was categorized as employed/self-employed, (coded '1'), or unemployed/out of the labor force (coded as '0'). The Income variable was measured, using equivalised 2005 household income after taxes. This was categorized into three groups of equal size, representing the high income (coded '1'), medium income (coded '2') and low income group (coded '3'). These variables were derived from administrative data from Statistics Norway and linked to the study sample.

*Bonding social capital *was measured by asking the extent of respondent's contact with immediate family members ('frequently in contact with family' coded '1' and 'not frequently in contact with family' coded '0') and friendship relations. This approach to measuring bonding social capital is in accordance with a notion of this form of resource as 'inward looking' networks consisting of people that are closely linked implying spouse, family and friends [32,33]. A similar approach to cover bonding social capital has been used previously [34].

*Bridging social capital *was measured firstly by determining the heterogeneity of networks in terms of whether both genders and people of different ethnic origin were represented. Thus, respondents were asked if they had friends with different ethnic background or of opposite gender (where positive cases were coded '1' and negative ones coded '0'). Secondly, we devised an indicator reflecting the scope of 'weak ties' in terms of acquaintances. Thus, we counted the number of acquaintances that the respondents reported to have among a predefined list of ten professional groups as diverse as farmer, craftsman, computer technician, shop steward, teacher, nurse, journalist, lawyer, doctor and politician. High scores on this variable are assumed to reflect a great scope and diversity in the respondent's social network. This operationalisation is in accordance with the definition of 'bridging social capital' as open networks that are 'outward looking' [33].

In assessing respondents' linking social capital we adopted a loose definition that considers contacts with resourceful persons. This type of capital was measured in two ways. Firstly, by asking whether the respondents were active members of any of five types of voluntary organizations (religious organizations, political organizations, sports club, resident's associations and 'other' type of organizations). The rationale behind sticking to those reporting to be *active *members was that mainly active members are likely to interact with other members to an extent creating network ties. In a Norwegian context, active membership of a voluntary organization is likely to link people to other resourceful persons. For instance, highly educated persons and persons from high income groups were reported to over-represent the active members of such organizations [35]. Using participation in organizations as indicator of this type of social capital is consistent with measurements used in a study that addressed the effect of different types of social capital on preventable hospitalization [36]. However, there are other studies that have used social organizations as a measurement of bonding social capital [37]. Thus, we devised two dummy variables, one for those reporting to be active in one organization (coded '1', else '0'), and one for those active in two or more organizations (coded '1', else '0'). Secondly, as a further indicator of linking social capital, we asked respondents if they counted a doctor and/or a nurse among their friends - assuming that having such health professionals in one's close network may link people to health improving resources ranging from information to various services (if person has a doctor as a friend it was coded '1', if else '0'. The idea was that in Norwegian context, health professionals, and doctors in particular, play an important role as 'gate-keepers' [38]. By socializing with such professionals one might learn which buttons to press to reach resources in the health system as well as the health related benefits and services in the social security system.

SPSS (Statistical Package for Social Sciences) version 16.0 was used for data analyses. Chi-square tests were used in calculating group differences. Before running the multivariate analyses, we performed a co-linearity diagnosis to make sure that independent variables were not related to each other. As the proportional odds assumption was violated we gave up ordinal logistic regression in favor of a generalized ordered logistic regression analysis to assess the associations between socioeconomic position and social network variables for self-rated health. First we examined impact of household income, ethnicity, age, gender, education and occupation on self reported health (model 1). Afterwards, social network variables were added into the model (model 2) to determine the importance of different types of network resources as well as the changes in odds of good health attributable to socioeconomic position variables when network is included in the model. Adjusted Odd ratio (OR) and 95% confidence interval (CI) were obtained for each variable. P-value < 0.05 was considered statistically significant.

## Results

Characteristics of study subjects are shown in table 2. The ratio of male to female was about 1:1. Two thirds of the study population were 40-67 years of age. The vast majority of the study population had either university education (35.8%) or secondary education (43.6%).

**Table 2:** Characteristics of the study population in relation to self-rated health

Characteristics	Poor health		Fair health		Good health		Total
	
	**No**.	%	**No**.	%	**No**.	%	**No**.
**Gender**							
Male	82	43.4	227	49.5	1227	49.7	1536
Female	107	56.6	232	50.5	1242	50.3	1581
**Marital relation**							
Cohabiting	117	62.2	336	73.8	1797	73.4	2250
Living alone	71	37.8	119	26.2	650	26.6	840
**Age**							
18-24	7	2.8	23	9.1	223	88.1	253
25-39	38	4.2	100	11.1	763	84.7	901
40-54	61	6	130	12.9	820	81.1	1011
55-67	55	7.9	153	21.9	492	70.3	700
≥ 68	28	11.1	53	21	171	67.9	252
**Ethnicity**							
Non-immigrants	172	91.5	410	89.5	2301	93.6	2883
Immigrants	16	8.5	48	10.5	158	6.4	222
**Occupation**							
In employment	73	38.6	281	61.2	2033	82.3	2387
Not employed	116	61.4	178	38.8	436	17.7	730
**Education**							
University	26	2.3	123	10.9	982	86.8	1131
Secondary	89	6.5	194	14.3	1076	79.2	1359
Primary/lower	68	12.3	128	23.1	359	64.7	555
**Income group**							
High income	36	3.6	107	10.6	871	85.9	1014
Medium income	60	5.9	148	14.6	805	79.5	1013
Low income	91	8.5	201	18.8	777	72.7	1069
**Frequency of contact with ones family**							
Yes	148	5.5	377	14	2163	80.5	2688
No	17	12.1	25	17.9	98	70	140
**Some one to talk to when distressed**							
Yes	152	5.9	355	13	2229	81.5	2736
No	32	14.3	77	27.8	168	60.6	277
**Gender diversity in one's social network**							
Yes	153	5.6	375	13.8	2181	80.5	2709
No	18	7.5	42	17.4	181	75.1	241
**Ethnic diversity in one's social network**							
Yes	78	6.5	158	13.2	965	80.3	1201
No	83	5.3	216	13.9	1257	80.8	1556

Of the 3,117 subjects that rated their health, 189 (6%) reported poor health, 459 (14.4%) rated their health fair and 2,469 (79.2%) rated their health good. Differences in self-rated health followed along socio-demographic lines. Thus, 61.4% (116) of those who were not in employment have rated their health poor. The corresponding proportion of those who were employed was 38.6% (73). This difference was statistically significant (P < 0.001). Significant differences (P < 0.001) in self-rated health was also observed among people with different educational levels, with good health becoming more prominent as level of education increases.

The ORs and 95% CIs for each variable are shown in table 3. In the regression model, the socio demographic variables showed mostly consistent associations with self-rated health: Age was negatively associated with good health while neither ethnicity nor gender was associated with self reported health in our analysis. The socioeconomic position indicators, household income, education and occupation were positively associated with good health, bearing witness to the social health inequalities in Norway. In this case, people who were employed or self-employed had almost three fold higher odds of self rated good health than those who were unemployed or out of the labour force. A similar tendency was observed when comparing those who had either primary or lower level of education and those who had university education (OR.3.15 CI. 2.00-4.96).

The significance of the association between good health and socioeconomic position was attenuated by adding social network variables into the model (model 2). This is especially the case concerning the impact of household income that was no longer statistically significant (p = 0.15). In line with expectations, several indicators of bonding social capital appear to be positively associated with good health. People who had someone to talk to when distressed had shown significantly higher odds of better health than those deprived from such an intimate person (p < 0.001 [OR: 2.17, CI: 1.55-3.02]). On the other hand, our indicators of bridging social capital did not turn out as associated with good health. The association between self-rated good health and heterogeneity of social network by gender did not show statistical significance. In contrast to expectations, having a network composed of people of different ethnic background is actually associated with less good health (p < 0.01 [OR: 0.56 CI: 0.36-0.86]). Considering indicators of linking social capital, people who were active in two or more voluntary organisations had significantly higher odds of reporting better health than those who were not active in any organisation (p < 0.001 [OR: 1.73 CI: 1.34-2.22]). Similarly, those who were active in one social organization had marginally higher odds of reporting better health than those who were not active in any organization - even if this difference failed to satisfy our criteria of statistical significance (p = 0.06). Counting a medical doctor among one's friends is in fact associated with better health (P < 0.02 [OR: 1.40 CI: 1.06-1.83]), while having a friend who is a nurse does not seem to have a similar impact.

**Table 3:** Associations between self-rated good health, SEP and social network factors

Variables	Socio-demographics alone (Model 1)		Social-network variables added (Model 2)	
	
	OR (95% CI)	P- value	OR (95% CI)	P-value
**Gender**				
Female	1.00			
Male	0.95 (0.76-1.14)	P = 0.621	0.86 (0.71-1.05)	P = 0.145
**Age**				
Age (18-74)	0.98 (0.97-0.99)	P < 0.001	0.98 (0.97-0.99)	P < 0.001
**Marital relation**				
Living alone			1.00	
Cohabiting			0.93 (0.75-1.16)	P = 0.551
**Ethnicity**				
Non-immigrant	1.00			
Immigrant	0.79 (0.57-1.08)	P = 0.151	1.00 (0.69-1.45)	P = 0.969
**Occupation**				
Not in employment	1.00		1.00	
Employed/self-empl.	2.85 (2.32-3.51)	P < 0.001	2.76 (2.23-3.52)	P < 0.001
**Education**				
No education/primary	1.00			
Secondary education	1.64 (1.31-2.04)	P < 0.001	1.51 (1.20-1.90)	P < 0.001
College education	2.40 (1.84-3.12)	P < 0.001	1.98 (1.49-2.62)	P < 0.001
University education	3.15 (2.00-4.96)	P < 0.001	2.62 (1.64-4.19)	P < 0.001
**Income**				
Low income group	1.00			
Medium income group	1.23 (0.99-1.54)	P = 0.05	1.18 (0.94-1.48)	P = 0.142
High income group	1.31 (1.03-1.66)	P < 0.03	1.20 (0.93-1.54)	P = 0.151
**Do you have a frequent contact with your family?**				
No			1.00	
Yes			1.25 (0.83-1.89)	P = 0.272
**Do you have someone that you can to talk to when distressed?**				
No			1.00	
Yes			2.17 (1.55-3.02)	P < 0.001
**Ethnic diversity in one's social network**				
No			1.00	
Yes			0.56 (0.36-0.86)	P < 0.01
**Gender diversity in one's social network**				
No			1.00	
Yes			0.89 (0.72-1.10)	P = 0.306
**Do you have a friend who is a nurse?**				
No			1.00	
Yes			1.12 (0.91-1.39)	P = 0.272
**Do you have a friend who is a medical doctor?**				
No			1.00	
Yes			1.40 (1.06-1.83)	P < 0.02
**Number of acquaintances reported among predefined list of ten prof. groups (0-10)**				
Acquaintances			1.00 (0.96-1.04)	P = 0.988
**Are you active member of a club/organization?**				
Not active member			1.00	
Active in 1 organization			1.22 (0.98-1.51)	P = 0.063
Active in ≥ 2 organizations			1.73 (1.34-2.22)	P < 0.001

## Discussion

The present study assessed the relationship between socioeconomic positions, three different types of social capital and self-assessed health of the adult population of Norway. There is a strong association between our indicators of socioeconomic position and health. However, this association is marginally attenuated when indicators of network resources are considered, indicating that the poor health of disadvantaged groups is partly related to poor access to different network resources. Other research has supported this interpretation [39,40].

In general, our findings support the result of prior studies that social networks are associated with self-rated health [4,22,27,41,42].

Under the *bonding *social capital heading, having an intimate person to talk to when distressed was among the most important factors predicting self-assessed good health for adult Norwegians. This is consistent with prior findings in the USA that family and friendship networks reduce distress and are important determinants of emotional wellbeing [5]. Epidemiological evidence shows that women without close relatives and friends before breast cancer was diagnosed had a 66% increased risk of all-cause mortality and a two-fold increased risk of mortality from breast cancer compared to their corresponding group [43]. This protective effect may be due to the social support provided by their social network, which may reduce the stress associated with having a potentially fatal disease. Frequency of contact with one's family was not significantly associated with self-rated good health in the present study. However, other studies found an association between contact with family and decreased morbidity and mortality [27,44]. In Norwegian context, the quality of the network (intimacy) and acquired support from family members may be more important than frequency of contact. However, our study did not investigate the acquired support.

Under the *bridging *social capital heading, diversity of network, indicated by whether or not both genders are represented, did not turn out to be significantly associated with self-assessed health for adult Norwegians. Likewise, networks where people of different ethnic backgrounds are represented were actually associated with having less good health in the present study. This might be explained by the fact that ethnic minorities in Norway are strongly overrepresented in exposed occupations like cleaning, retail, transportation and manual jobs in general [45]. Thus, those who have networks where ethnic minorities are represented are themselves more likely to be working in such types of occupations. Despite the apparent lack of association between *bridging *social network variables and self-rated good health in our analysis, the importance of *bridging *social capital for good health should not be underrated. Other research has associated diverse network with better prognoses for those who face life-threatening chronic illnesses. For example, research shows that more socially diverse people with larger social circles develop less risk of coronary artery disease and live longer [6]. A prior study by Putnam [33] concludes that the more socially diverse people are in the community, the less likely that they suffer from colds, heart attacks, strokes, cancers, depression, and premature deaths of all sorts. In our study, only 7% of our respondents had a non-Norwegian ethnic background and this may explain the divergence of our findings with previous findings. On the other hand, this study didn't find significant association between number of acquaintances and self-reported good health. Prior study suggested that visits to acquaintances and friends may provide entertainments and socialization leading to reduction of stress [46]. However, our finding is consistent with other studies that found no difference in the number of acquaintances between people with mental problems and people with no mental problems [47,48].

Besides, under the *linking *social capital heading, people who were active members of two or more social organizations had greater odds (OR: 1.73, CI: 1.34-2.22) of good health than those who were not members of any social organization. In our study, a significantly higher proportion (38.8%) of people ≥ 68 yrs reported poor health if they were not a member of a social organization compared to those <25 yrs (14%). In Norway, where there has been a considerable increase in the proportion of elderly people during the last decades, social engagement may be an important aid in promoting good health in later life. A study among older people in Ireland associated social engagement with better quality of life, self-rated happiness and the view that life is worth living [17]. The role of social network is crucial for older people, who often experience social transitions, such as retirement, and the inability to participate in social activities because of a disability or the lack of mobility [49]. The forthcoming message from this result is that older people need special attention and may benefit from interventions that promote social interaction. While social network is exceptionally important for the health and well-being of older people, a prior study in South Korea found that social participation is important for the health of all age groups and both genders [50]. In recognition of this fact, we suggest a national health care plan that recognizes the significance of social network and that promotes the maintenance of social connections across the life span of individuals. Our study shows that employment and education is significantly associated with self-rated good health. It has been widely reported that social network is closely associated with SEP due to the fact that people with low SEP have lower social network and support [51,52]. However, the association between income and self-rated health disappeared after we added social network variables into the model. Evidence shows that health interventions to increase the social interaction and cohesion in a community are as worthwhile as improved access to medical care or the routine provision of medical care [53]. Social participation provides people with emotional support, self- fulfillment and information about healthy lifestyles, while protecting them from the adverse effects of loneliness [4,54]. The current Norwegian health strategy to reduce social inequality in health focuses upon individual health risks, the health of specific disadvantaged groups, and the possible health implications of poverty [55]. Our findings suggest that supplementing the current strategy with measures that promote social network formation and social participation that link disadvantaged individuals to people from other strata may reduce the social inequality in health in Norway.

Among the weaknesses of this study is the fact that our data was cross-sectional and consequently does not allow for inferences regarding causation. There is a possibility of reverse causality, i.e. decreased social network resulting from poor health. In particular, it is likely that poor health hampers people's ability to be active members in clubs and organizations - and especially so when it comes to sports associations. In general, there may be reasons to believe that the observed association between social network and self-assessed health is a mixture of causal effects in both directions. Thus, further prospective studies are required to eliminate the possible effects of reverse causality. This might be accomplished by assessing social characteristics and then by following-up people to measure subsequent changes in health while controlling for baseline health. Furthermore, the group of disadvantaged persons seems to be underrepresented in our sample. However, we considered the indicators of socioeconomic position and demographics to be a sufficient control in the multivariate analyses.

## Conclusion

Our study found that deficits in social networks are predictive of self-rated poor health. The study suggests that part of the association between socioeconomic position and health is explained by the differences in access to network resources. The association between network and health is particularly pronounced when it comes to types of social network accommodated by the notion of *bonding social capital *(referring to close and strong social ties). However, this association is also found when it comes to indicators of network resources accommodated by the notion of *linking social capital *(referring to active participation in clubs and associations and whether one counts a doctor as a friend). This study did not find association between frequency of contact with family members and number of acquaintances, respectively, and self-reported health.

Based on our findings, we suggest that, a national strategy aimed at reducing social health inequalities should consider measures to promote social network formation and social participation among disadvantaged groups. In recent years there have been some initiatives aiming at promoting social participation of such groups. For instance, in Norway, a so-called Network Conference Program has been developed for long-term social assistance claimants. This program aims at facilitating participants' network resources by bringing together potentially important persons of their choice in a conference to discuss how to obtain certain important outcomes in participants' life. The effect of the intervention including health effects are currently being evaluated by means of an RCT.

## Competing interests

The authors declare that they have no competing interests.

## Authors' contributions

AG: Did data analyses and drafted the manuscript. IH: Was involved in the data collection, data analyses and in writing the manuscript. All authors read and agreed the final draft of the manuscript.
